# Global research prospects and trends in TFH cells and tumors: a bibliometric analysis

**DOI:** 10.3389/fonc.2025.1443890

**Published:** 2025-02-14

**Authors:** Hao Lei, Jin Hu, Junpeng Zhu, Runze Li, Yu Zhao, Yaqi Zhao, Guisheng He, Tao Song, Chong Lu, Wuping Zheng, Lei Li, Chunping Liu, Hengyu Chen

**Affiliations:** ^1^ Department of Breast and Thyroid Surgery, The Second Affiliated Hospital of Hainan Medical University, Haikou, China; ^2^ Department of Breast and Thyroid Surgery, Renmin Hospital of Wuhan University, Wuhan, China; ^3^ Department of Breast and Thyroid Surgery, Union Hospital, Tongji Medical College, Huazhong University of Science and Technology, Wuhan, China

**Keywords:** Tfh cells, tumor, immune, bibliometrics, CD4+Th cells

## Abstract

**Background:**

T follicular helper (TFH) cells, a subset of CD4^+^Th cells, play a critical role in B cell activation, proliferation, and differentiation primarily within B follicles in secondary lymphoid organs, essential processes for effective antibody responses. TFH cells are also implicated in various conditions, including autoimmune diseases, cancer, infectious diseases, allergies, and vaccine reactions. Despite their broad impact, a review of the literature on TFH cells and tumors has not been conducted. We aimed to fill this gap by providing a detailed analysis of the research landscape concerning TFH cells and tumors.

**Method:**

We conducted a bibliometric analysis of literature on TFH cells and tumors from 2012 to 2024 using the Web of Science Core Collection (WoSCC). For an analysis of the global research landscape, we employed VOSviewer (version 1.6.20), CiteSpace 6.2.R6 software, and the “bibliometric” package in R language (version 4.3.2) to evaluate data on countries/regions, authors and cited authors, institutions, journals, references, and keywords. We also conducted a systematic review to summarize the global research trends, prospects, and hotspots in this field.

**Results:**

Our analysis included contributions from 60 countries/regions, 7,864 authors, 35,853 cited authors, 1,756 institutions, 385 academic journals, 50883 references, 222 keywords, and 1,181published papers. Over the past decade, the volume of research on TFH cells and tumors had consistently increased. China published the most papers, more than double that of the United States. The top 2 authors ranked by publication volume were Gaulard, Philippe (14 articles, 379 citations), and De leval, Laurence (12 articles, 236 citations) Notably, 9 of the top 10 most published institutions were from China. Frontiers in Immunology and Immunity were the leading journals in publications and citations. A cluster analysis revealed a shift in research focus from “expression”,”B cells” and “survival” to “tumor microenvironment”, “tumor infiltrating immune cells” and “immune infiltration” in recent years.

**Conclusion:**

This bibliometric analysis suggests that TFH cells hold significant research value and potential clinical applications in tumor immunotherapy. Moreover, the bibliometric analysis offers valuable references and guidance for related research endeavors. It also points out the prevailing issues and challenges in TFH cell research, and underscores the need for further basic and clinical research to advance the related fields.

## 1 Introduction

Follicular helper (TFH) cells, a distinct subset of T cells characterized by unique transcriptional profiles and functions, has emerged as a novel cell type over the last decade. At present, TFH cells are defined as CD4^+^ CXCR5^+^ PD-1^+^T cells, including three subgroups: TFH1 (CD3 ^+^ CD4 ^+^ CD45RA^-^ CXCR5 ^+^ CXCR3 ^+^ CCR6^-^), TFH2 (CD3^+^ CD4^+^ CD45RA^-^ CXCR5^+^ CXCR3^-^ CCR6^-^), and TFH17 (CD3^+^ CD4^+^ CD45RA^-^ CXCR5^+^ CXCR3^-^ CCR6^+^) (1). First identified in human tonsils in 2000 and 2001, TFH cells are noted for their high expression of CXCR5 (2), and depend on the transcription factor Bcl6 for their function (3–6). The interaction between TFH cells and B cells, promotes B cell proliferation, class switching and affinity maturation. Class switching recombination (CSR) is a process that occurs after B cell activation, allowing B cells to change the type of antibodies they produce, such as converting from IgM to IgG, IgA, or IgE. The cytokines produced by TFH cells, such as IL-4 and IL-21, are crucial for CSR. In particular, TFH1 and TFH2 cells play a crucial role in isotype conversion, with TFH1 cell deficiency leading to reduced IgG2c and IgG2a conversion, while sustained IL-4 production by TFH2 cells can drive IgE conversion (7). Somatic hypermutation (SHM) is another process that B cells undergo in the germinal center (GC), involving mutations in the mutated region of the B cell receptor (BCR) gene, which can increase the affinity of BCR for antigens. TFH cells are crucial for SHM by providing co stimulatory signals and cytokines such as IL-21. IL-21 is absolutely essential for plasma cell formation and SHM (8). And gene conversion is another mechanism by which B cells generate diversity, involving the insertion of additional DNA fragments after V (D) J recombination, which can further increase antibody diversity. Although the role of gene conversion in B cell development is not as widely studied as in SHM and CSR, TFH cells may indirectly affect this process through the cytokines they produce.Recent research by Li Hanjie’s team has revealed that inhibiting the formation of tertiary lymphoid structures (TLS) through TFH or B cell depletion during the invasion of lung adenocarcinoma (LUAD) can promote tumor growth in mouse models. The anti-tumor effect of TFH dependent TLS is mediated through interleukin 21 (IL-21) - IL-21 receptor signaling (9). TFH cells play a crucial role in germinal center formation, influencing the differentiation of germinal center B cells into plasma cells and memory B cells (3, 6, 10). TFH cells also exhibit significant expression of IL-21 (10, 11). While the important role of TFH cells in infection and vaccination has been elucidated, their involvement in cancer is a burgeoning area of research. In malignancies originating from TFH cells or associated with B cells, an elevated TFH cell count often correlates with poor prognosis. Conversely, their presence in various non-lymphocytic solid tumors is frequently linked to a more favorable prognosis (12).

Bibliometric analysis is a quantitative method to delineate the knowledge structure and developmental trends within a specific field, assessing research output, productivity, and impact. This method, unlike other major review methodologies, is particularly well-suited for an evaluation of entire disciplines, encompassing thousands of publications. It offers a robust framework for evaluating the impact of scientific publications through mathematical and statistical techniques, thus identifying research gaps and areas that require further investigation (13). Despite the growing body of literature on the relationship between TFH cells and tumors in recent years, bibliometric studies on this topic remain scarce. Our study aimed to address this gap by conducting a bibliometric network analysis to assess the structural framework, current landscape, and future trajectories of research on TFH cells and tumors.

This bibliometric analysis focuses on papers concerning TFH cells and tumors published from January 1, 2012 to December 31, 2024, resulting in the retrieval of 1,181 articles. A bibliometric analysis was then performed to pinpoint the current research trends.

## 2 Materials and methods

### 2.1 Data acquisition and search strategy

**Figure 1:**
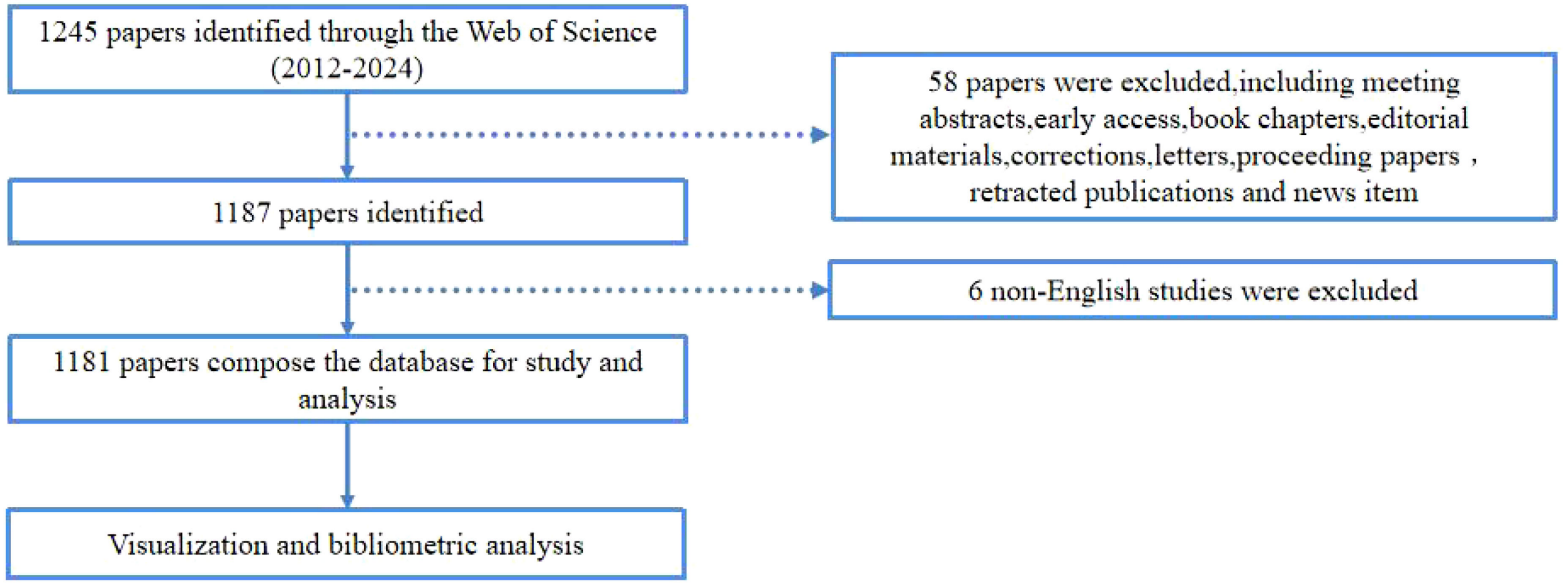
The flowchart illustrating the search strategy and selection process in TFH cell and tumor.

The Web of Science (WoS) database is widely acknowledged for its reliability and comprehensive coverage of academic information, making it the preferred choice for bibliometric analysis. A database for bibliometric analysis was established by retrieving relevant literature from the WoS Core Collection database. To ensure data accuracy and consistency, especially considering potential database upgrades, we conducted a thorough search and data export on January 9, 2025, encompassing published articles on TFH cells and tumors from 2012 to 2024. The search strategy included the terms: (TS = (T follicular Helper Cells) OR TS = (follicular B Helper T Cells) OR TS = (follicular Helper T Cells) OR TS=(TFH Cell) OR TS = (TFH Cells) AND (TS = (Tumor) OR TS = (Neoplasms) OR TS = (Neoplasia) OR TS = (Cancer)). To minimize potential biases, specific refinement criteria were applied: (1) only articles and reviews were included; (2) language was restricted to English; (3) the timeframe was set from January 1, 2012, to December 31, 2024. A total of 1,181 articles were retrieved for detailed analysis. The details of literature screening are summarized in **Figure 1**.

### 2.2 Data analysis

In this study, we utilized the “bibliometric” package within R (version 4.2.3) for in-depth scientific mapping analysis (8). We employed two distinct software tools for bibliometric analysis: CiteSpace [6.2.R6] and VOSviewer (version 1.6.20). CiteSpace, a Java-based citation visualization software developed by Chaomei Chen, facilitates statistical analysis and transforms raw data into visual representations of literature networks (14). Through CiteSpace, we examined keywords and references with significant citation bursts. VOSviewer, a robust bibliometric surveying and mapping tool developed by Nees Jan van Eck and LudoWaltman in 2009 (15), was used to visualize authors, cited authors, and institutions. Our study undertook a thorough and systematic evaluation of the research field by integrating these two software tools.

## 3 Results

### 3.1 Temporal distribution of the literature

**Figure 2:**
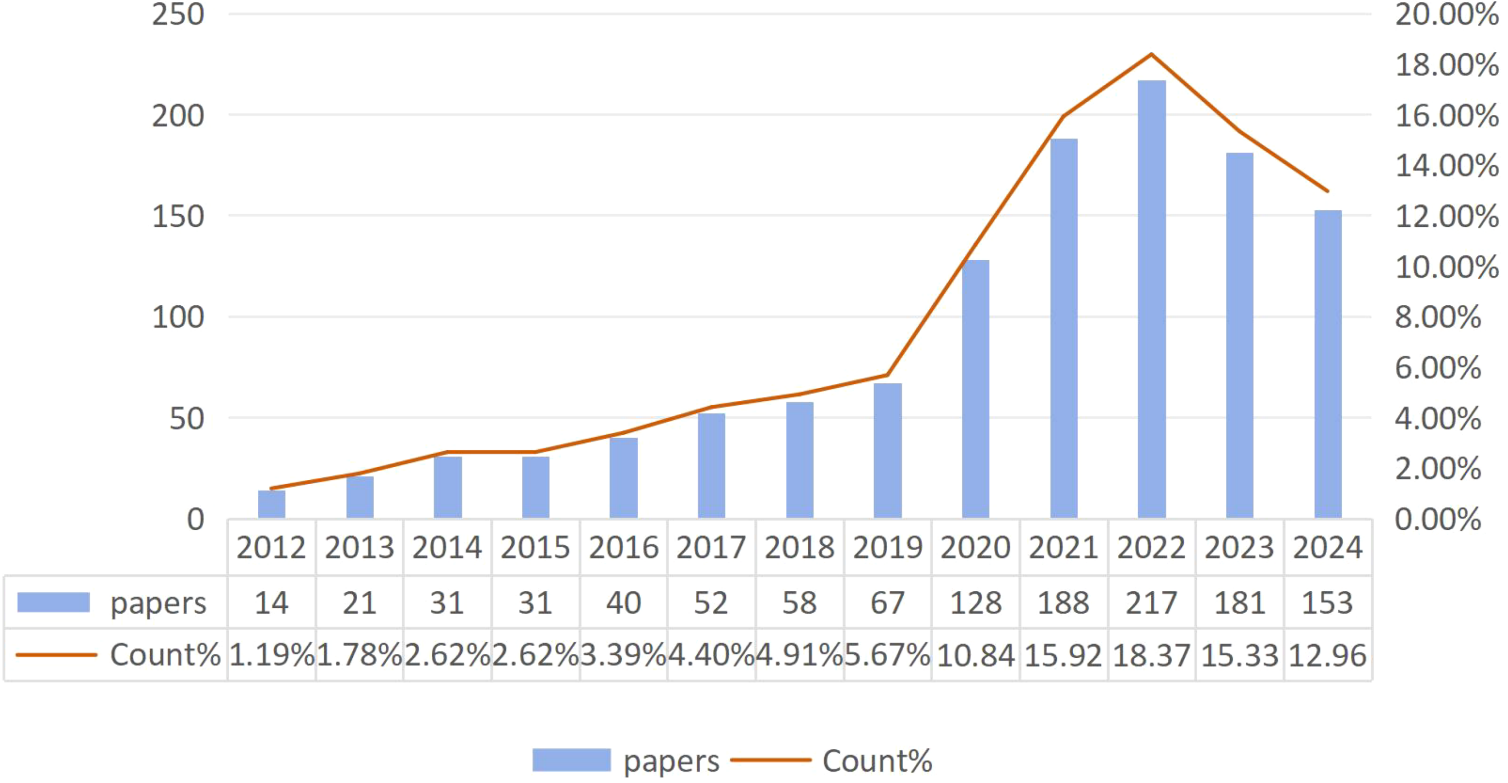
Trends in the growth of publications.

A total of 1,181 papers on TFH cell and tumor were reviewed, comprising 986 articles and 195 reviews. **Figure 2** illustrates a fluctuating increasing trend in publications since 2012, indicating a growing research interest in this field. Notably, in the last five years, the number of publications had more than doubled the total publications of previous years. The year with the lowest publication count was 2012 (14 articles, 1.19%), and the year with the highest was 2022 (217 articles, 18.37%).

### 3.2 Distribution of countries/regions

**Figure 3:**
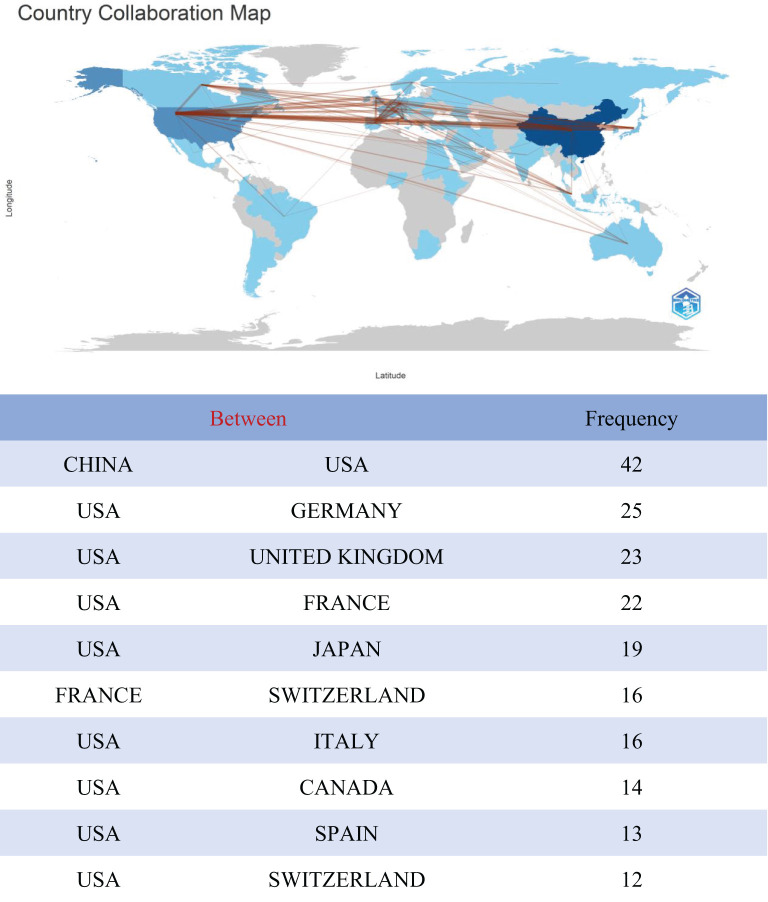
Analysis of the collaborative network between TFH cells and tumor related literature sources in different countries.

Papers on TFH cells and tumors had been published across 60 countries or regions globally. The top 10 countries in terms of publication volume are presented in **Table 1**. Leading in publication volume were China (624 articles, 52.84%), the United States (304 articles, 25.74%), and France (71 articles, 6.01%). Together, China and the United States accounted for nearly 70% of global publications, with China alone accounting for almost half of these publications. However, the United States has a citation count of 15,137 and an H-index value of 60, which is higher than that of China. A cross-border cooperation map illustrated the collaboration density between countries, with notable close collaboration between China and the United States, which had partnered in 42 times (**Figure 3**). The United States not only published a substantial volume of articles but had also engaged in extensive international cooperation. It had forged deep collaborative relationships with countries such as China, Germany, the United Kingdom, Japan, France, and Italy. This approach set a valuable role model for other nations to follow.

**Table 1:** Metrics of publications from the top 10 countries in the area of TFH cells and tumor research.

Top 10	Country	Publications	Count%	H-index	Times Cited
1	CHINA	624	52.84%	43	9571
2	USA	304	25.74%	60	15137
3	FRANCE	71	6.01%	27	5342
4	JAPAN	71	6.01%	22	1837
5	GERMANY	68	5.76%	28	6197
6	ENGLAND	52	4.40%	27	3423
7	ITALY	44	3.73%	20	1983
8	SWITZERLAND	38	3.22%	17	1463
9	CANADA	37	3.13%	17	1609
10	SPAIN	34	2.88%	16	1525

### 3.3 Authors and co-cited authors

**Figure 4:**
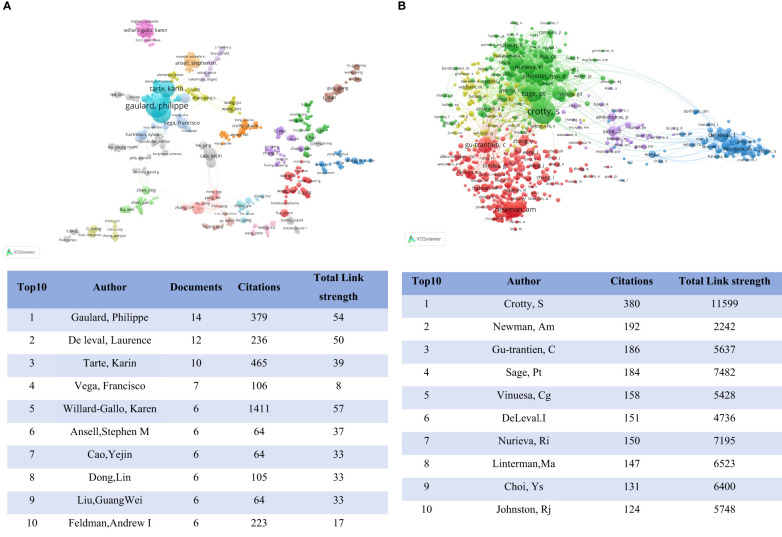
**(A)** Author visualization: Node size represents the number of articles. **(B)** Co-citation author analysis chart, where the size of nodes represents the number of repetitions.

Since 2012, a total of 7,864 authors and 35,853 cited authors had contributed to the field of TFH cells and tumors. The author visualization diagram (**Figure 4A**) illustrates collaboration among co-authors and the publication output of each author. A threshold of at least 2 articles per author was applied, resulting in the inclusion of 767 authors in the network. The top 3 authors based on publication count were Gaulard, Philippe (14 articles, 379 citations), De Leval, Laurence (12 articles, 236 citations), and Tarte, Karin (10 articles, 465 citations). In the co-citation analysis (**Figure 4B**), a minimum citation threshold of 12 per author was applied, leading to the inclusion of 734 authors in the network. The most frequently cited authors were Crotty, S (380 citations), Newman, Am (192 citations), and Gu-Trantien, C (186 citations). These results suggested a notable interest among these authors in the study of TFH cells and tumors.

### 3.4 Analysis of the most productive institutions

**Figure 5:**
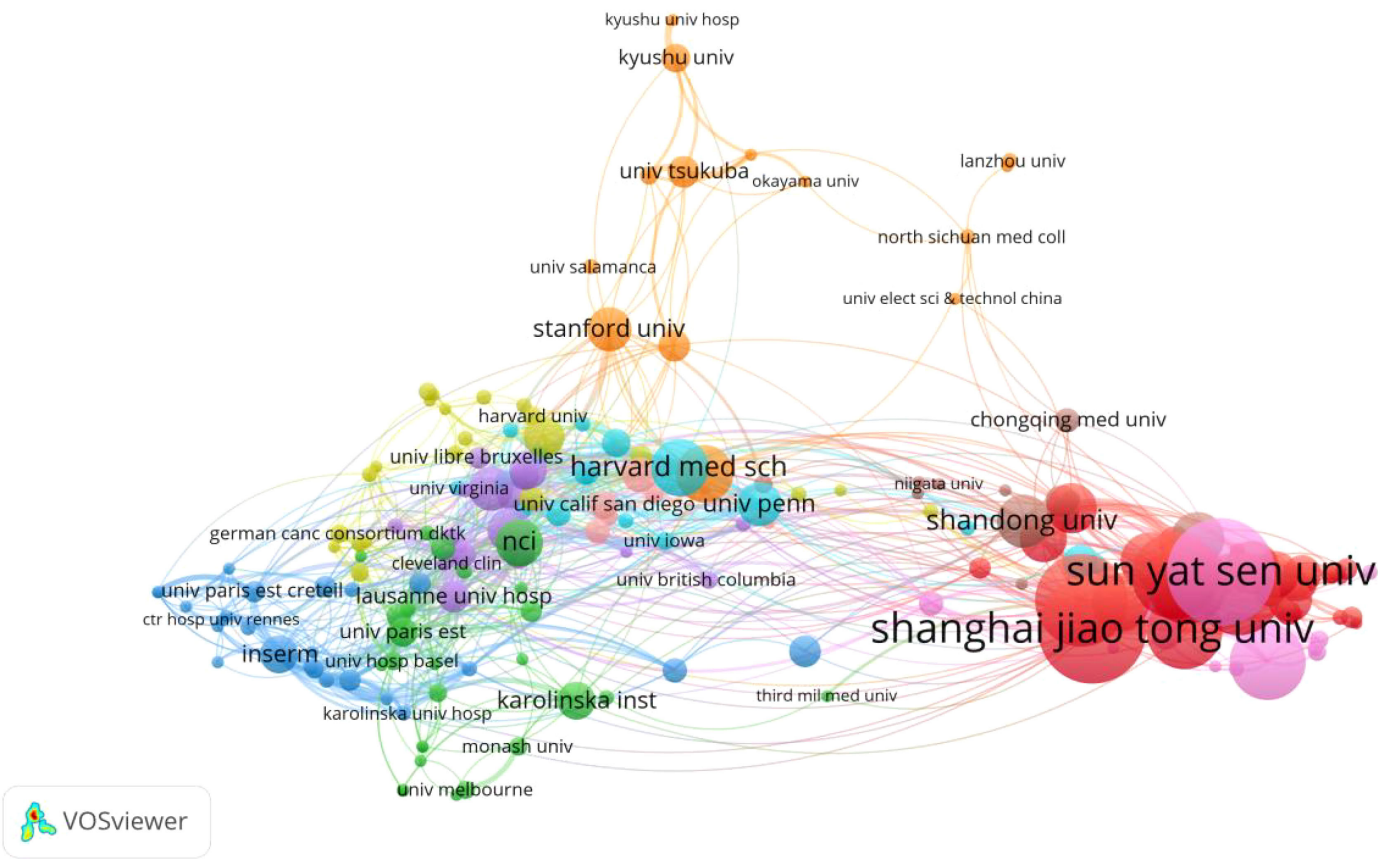
Institutional co-occurrence chart.

A total of 1,756 institutions had engaged in research on TFH cells and tumors. Among them, the top 10 institutions collectively published 265 articles, representing22.4% of the total publications (**Table 2**). Leading in publication volume were Shanghai Jiao Tong University (34 articles, Citations:887), Sun Yat Sen University (34 articles, Citations:454), and Fudan University (29 articles, Citations:567). Notably, The top 10 institutions are all located in China, underscoring the country’s prominent position in this field. Then, we used VOSviewer to visualize the density map of the extended network and inter-institutional collaborations (**Figure 5**). The node sizes reflected the number of articles per institution, and the curve thickness indicated the strength of collaboration. Different colors on the map represented distinct collaboration groups. Notably, Sun Yat-sen University, Shanghai Jiao Tong University, and Fudan University exhibited extensive connections with other institutions and were positioned at the core of the density map.

**Table 2:** Top 10 institutions with the highest number of publications.

Top 10	Organization	Articles	Citations	Country
1	Shanghai Jiao Tong University	34	887	China
2	Sun Yat-sen University	34	454	China
3	Fudan University	29	567	China
4	Huazhong University of Science and Technology	27	307	China
5	Zhejiang University	26	1041	China
6	Central South University	26	435	China
7	Southern Medical University	24	234	China
8	Nanjing Medical University	24	307	China
9	Tongji University	21	625	China
10	Fujian Medical University	20	104	China

### 3.5 Analysis of the higher-impact journals

A total of 385 journals had published articles on TFH cells and tumors, with **Table 3** listing the top 10 most prolific journals in this field. Notably, In the JCR (2023) partition, all these journals are in Q2 or above, underscoring their substantial academic influence. Together, these top 10 journals accounted for 24.8% of all publications on this topic. FRONTIERS IN IMMUNOLOGY had published 96 articles, making it the journal with the largest circulation. It was followed by FRONTIERS IN ONCOLOGY with 41 articles, and FRONTIERS IN GENETICS with 28 articles. The top 3 journals with the highest citations were IMMUNITY (cited 3,346 times), J IMMUNOL (cited 3,322 times), and BLOOD (cited 3,081 times), as detailed in **Table 4**.

**Table 3:** The top ten journals with the most sources of literature.

Top 10	Sources	Articles	IF (2023)	JCR
1	FRONTIERS IN IMMUNOLOGY	96	5.7	Q1
2	FRONTIERS IN ONCOLOGY	41	3.5	Q2
3	FRONTIERS IN GENETICS	28	2.8	Q2
4	CANCERS	24	4.5	Q1
5	SCIENTIFIC REPORTS	23	3.8	Q1
6	INTERNATIONAL IMMUNOPHARMACOLOGY	20	4.8	Q1
7	BLOOD	16	21	Q1
8	FRONTIERS IN CELL AND DEVELOPMENTAL BIOLOGY	15	4.6	Q1
9	INTERNATIONAL JOURNAL OF MOLECULAR SCIENCES	15	4.9	Q1
10	JOURNAL OF IMMUNOLOGY	15	3.6	Q2

**Table 4:** The top ten journals with the most citations.

Top 10	Sources	Total citations	IF(2022)	JCR
1	IMMUNITY	3346	25.5	Q1
2	J IMMUNOL	3322	3.6	Q2
3	BLOOD	3081	21	Q1
4	NATURE	2226	50.5	Q1
5	J EXP MED	2208	12.6	Q1
6	NAT IMMUNOL	2014	30.5	Q1
7	FRONT IMMUNOL	1467	5.7	Q1
8	SCIENCE	1451	44.7	Q1
9	P NATL ACAD SCI USA	1377	9.4	Q1
10	CELL	1368	45.5	Q1

### 3.6 Co-cited references and references bursts

**Figure 6:**
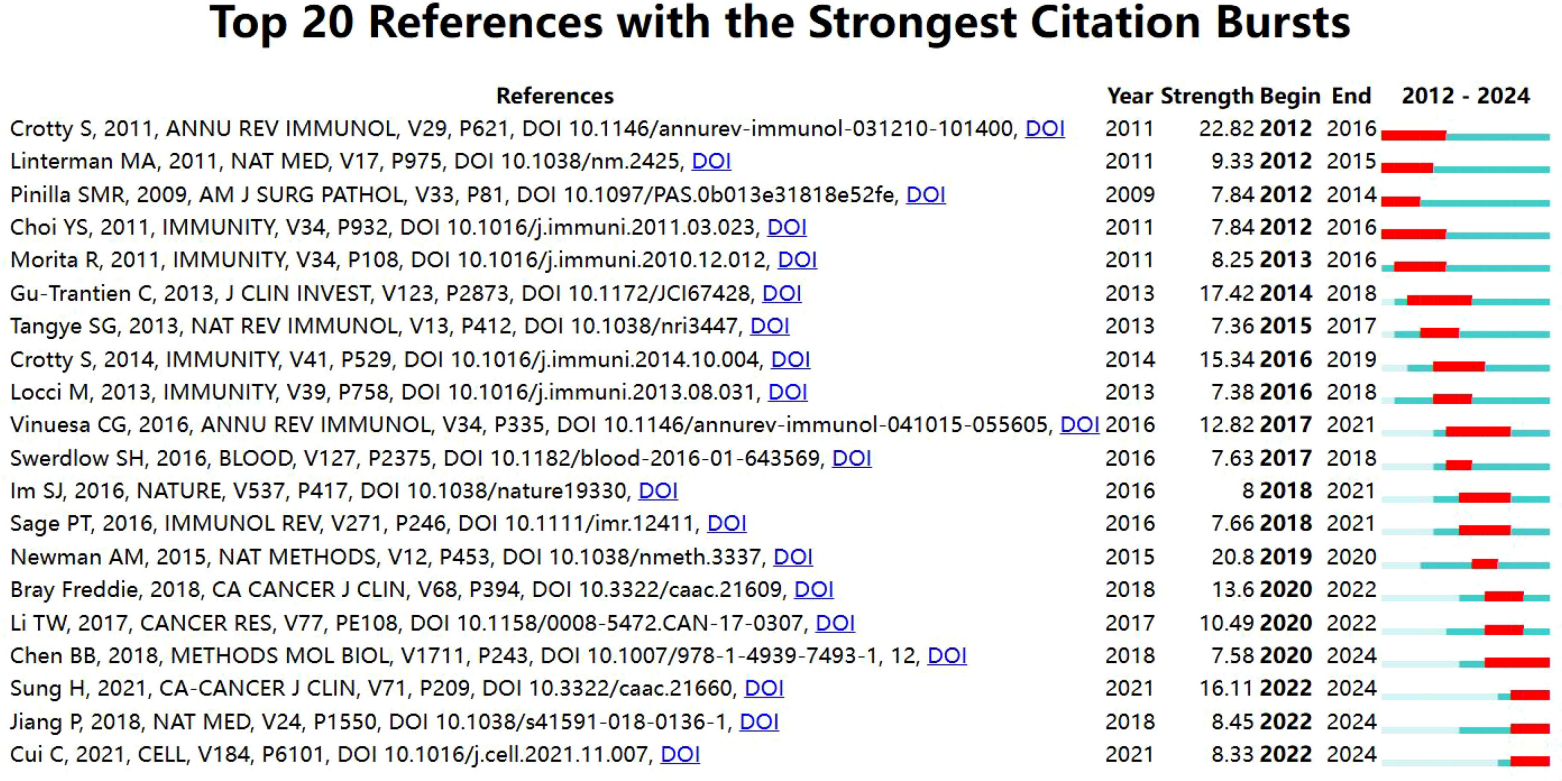
Cite the top 10 most explosive references.

In this study, among the 50,883 retrieved references, the top 10 most cited papers collectively received 10,513 citations. **Table 5** presents these references in descending order of citation frequency, each having garnered over 200 citations. The top 3 most cited articles were:Spatiotemporal dynamics of intratumoral immune cells reveal the immune landscape in human cancer (cited 3,721 times); T follicular helper cell differentiation, function, and roles in disease (cited 1,842 times); and T Follicular Helper Cell Biology: A Decade of Discovery and Diseases (cited 1,362 times). An analysis of these highly cited articles (**Figure 6**) provided insights into the latest advancements in the field of TFH cells and tumors. Notably, the article “Follicle helper CD4 T cells (TFH)” published in Annu Rev Immunol had the highest citation explosion value from 2012 to 2016, at 22.82.

**Table 5:** The top ten most cited articles.

Top 10	Cited References	Journal/year	Citations
1	Spatiotemporal dynamics of intratumoral immune cells reveal the immune landscape in human cancer	Immunity/2013	3721
2	T follicular helper cell differentiation, function, and roles in disease	Immunity/2014	1842
3	T Follicular Helper Cell Biology: A Decade of Discovery and Diseases	Immunity/2019	1362
4	CD4^+^ follicular helper T cell infiltration predicts breast cancer survival	J Clin Invest/2013	1115
5	The gene expression profile of nodal peripheral T-cell lymphoma demonstrates a molecular link between angioimmunoblastic T-cell lymphoma (AITL) and follicular helper T (TFH) cells	Blood/2006	699
6	Recurrent TET2 mutations in peripheral T-cell lymphomas correlate with TFH-like features and adverse clinical parameters	Blood/2012	513
7	B Cells and T Follicular Helper Cells Mediate Response to Checkpoint Inhibitors in High Mutation Burden Mouse Models of Breast Cancer	Cell/2019	392
8	Activating mutations in genes related to TCR signaling in angioimmunoblastic and other follicular helper T-cell-derived lymphomas	Blood/2016	332
9	CXCL13-producing TFH cells link immune suppression and adaptive memory in human breast cancer	JCI Insight/2017	331
10	Characterization of intratumoral follicular helper T cells in follicular lymphoma: role in the survival of malignant B cells	Leukemia/2011	206

### 3.7 Analysis of keyword co-occurrence

**Figure 7:**
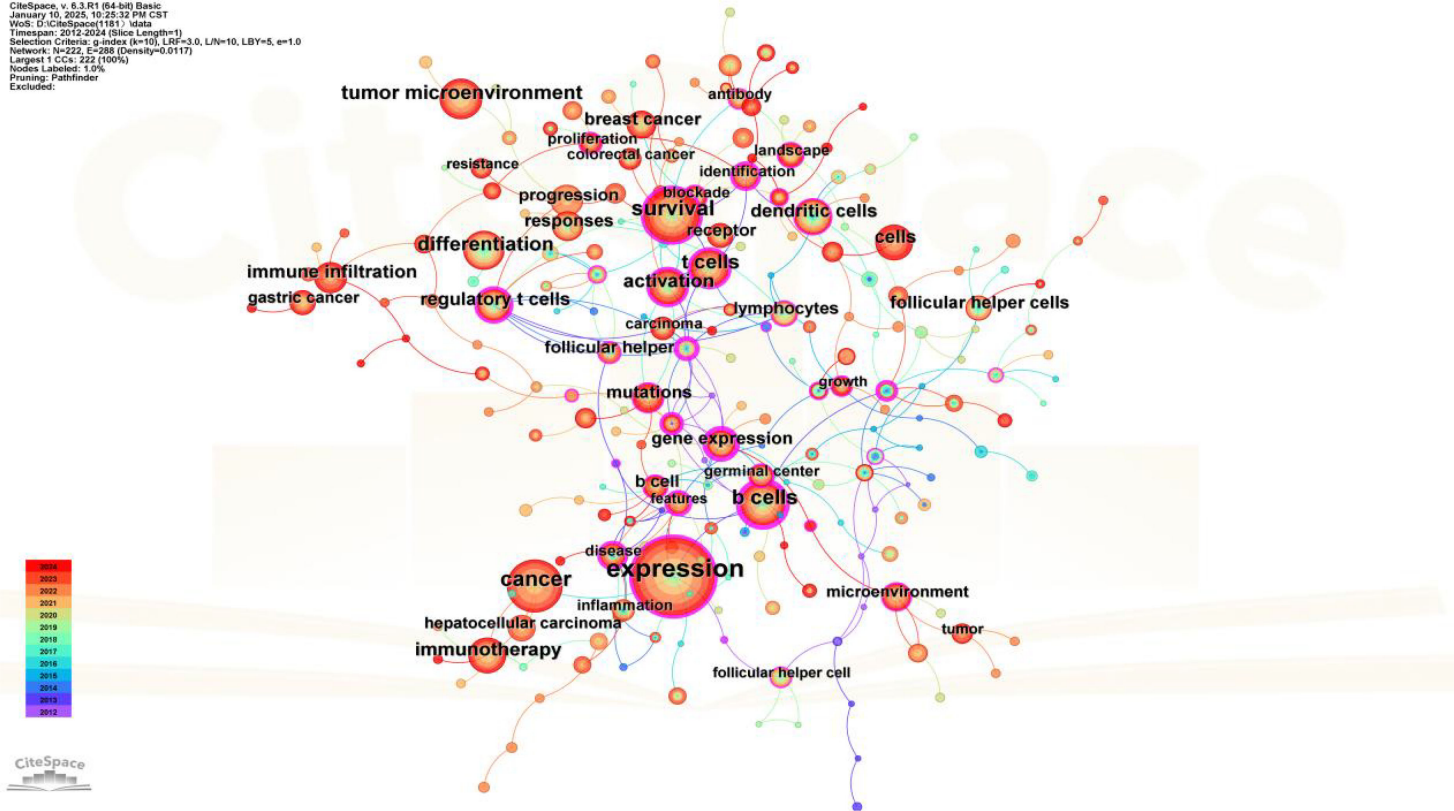
Keyword co-occurrence.

**Figure 8:**
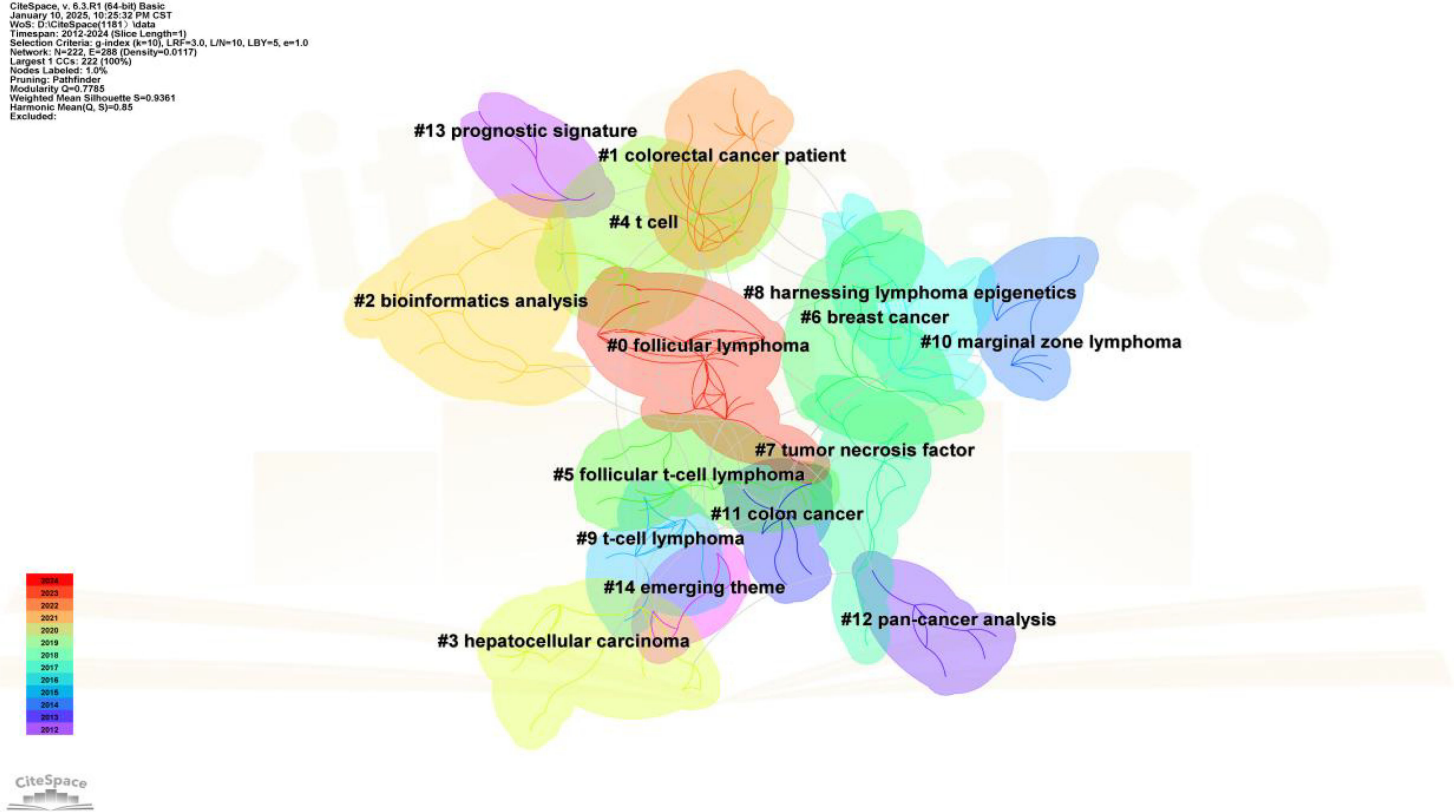
Keyword clustering.

**Figure 9:**
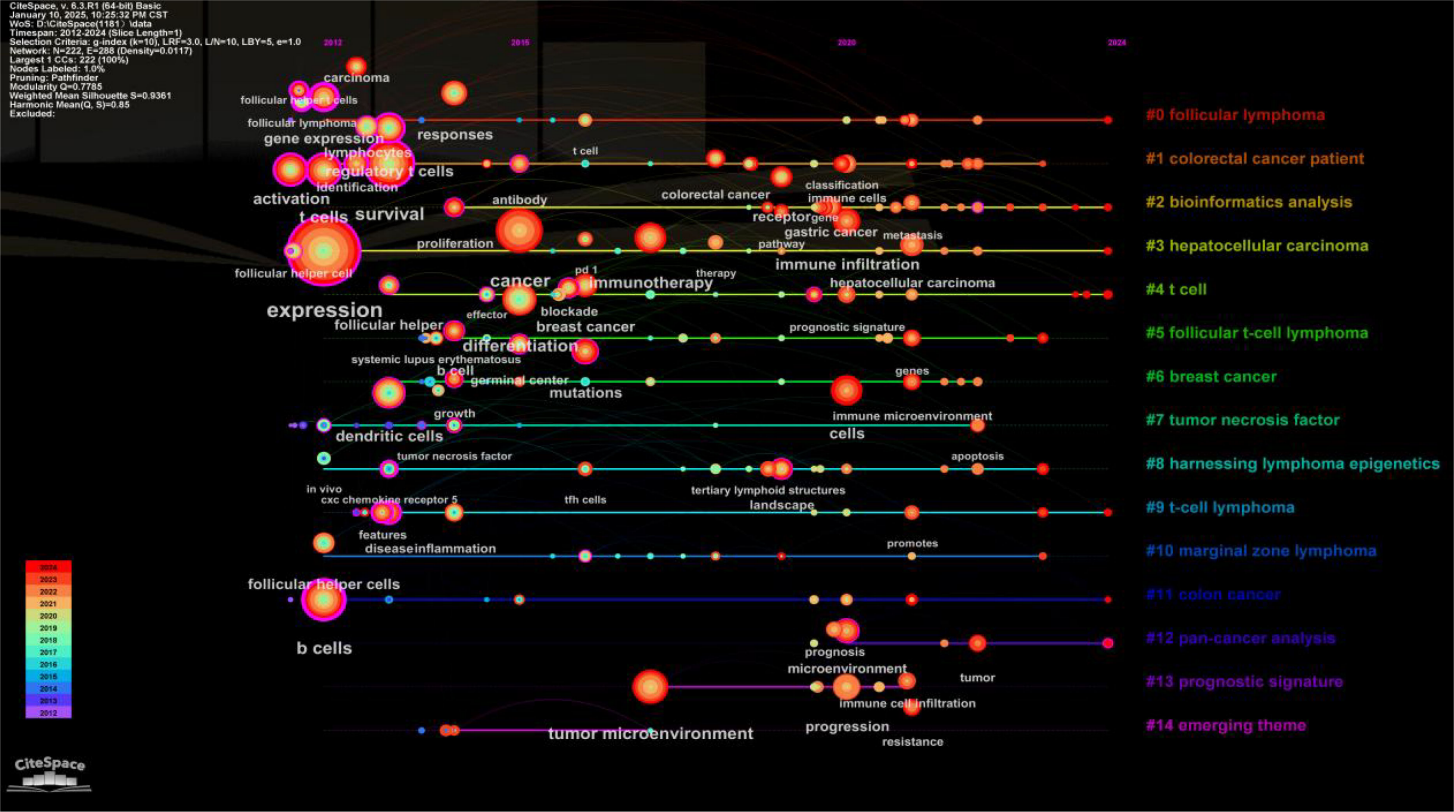
Keyword timeline.

**Figure 10:**
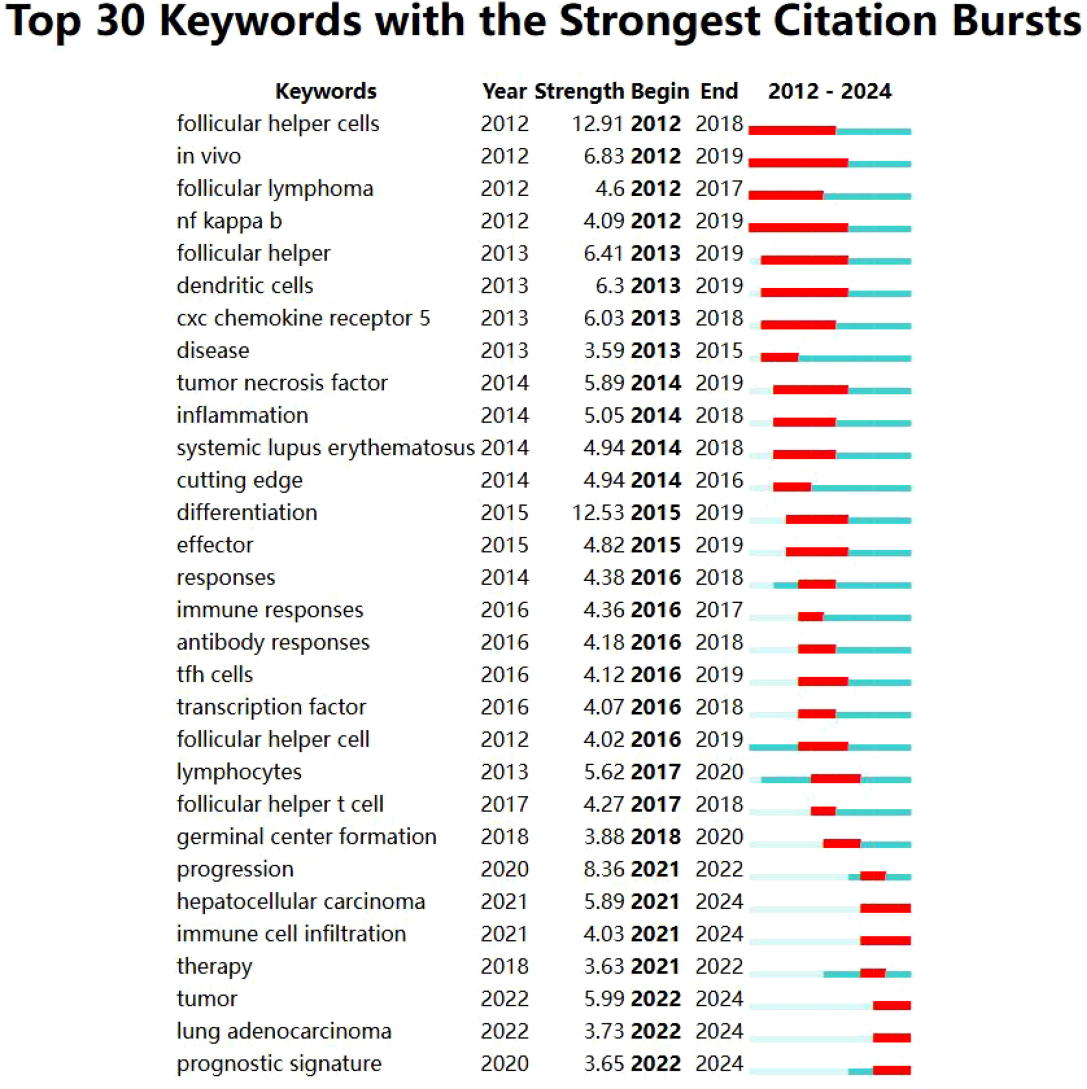
Keyword emergence chart.

Keyword co-occurrence analysis is valuable for uncovering semantic relationships between keywords, extracting latent information from text data, and enhancing comprehension of text content and associated themes. Employing CiteSpace to visualize the keyword co-occurrence network (**Figure 7**). The most frequently appearing keywords were “expression” (366 occurrences), “cancer” (162 occurrences), “survival” (142 occurrences), “B cells” (119 occurrences), and “differentiation” (105 occurrences). Subsequent cluster analysis (**Figure 8**) revealed 15 primary categories focused on research areas such as TFH cells and tumors. These categories included topics like “follicular lymphoma,” “colorectal cancer patient,” “bioinformatics analysis,” and “hepatocellular carcinoma.” To visually illustrate the temporal dynamics of keywords, we generated a keyword timeline chart (**Figure 9**) and a keyword emergence chart (**Figure 10**). The evolution of these keywords reflected the shifting priorities over time, indicating both continuity and variability in research focus. Recent trends showed a transition towards topics like “tumor microenvironment”, “tumor infiltrating immune cells”, and “immune infiltration”. This transition indicated the ongoing development and maturation in the field. Initially, research emphasis was on “expression,” “B cells,” and “survival,” with a gradual shift towards emerging areas of interest in recent years.

**Figure 11:**
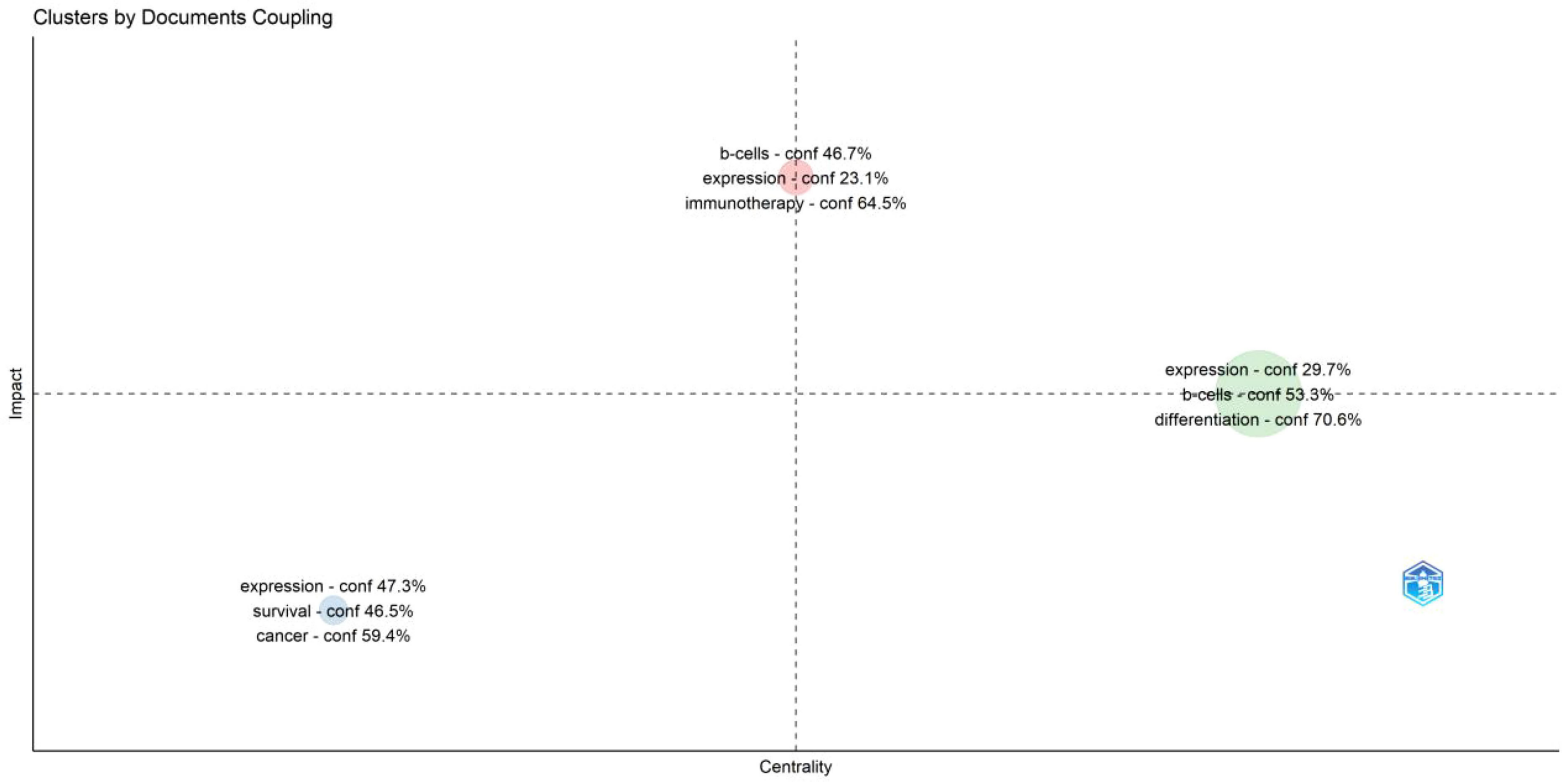
Theme distribution map.

Theme analysis examined author’s keywords and their connections to identify central theme. In a thematic network, nodes with more connections were considered more central and pivotal. The cohesion among nodes, reflecting the density of the research field, indicated their capacity for growth and sustainability. We presented a thematic map of TFH cells and tumor research, where the theme “immunotherapy” in the first quadrant (upper right corner) indicated both significant and robust development (**Figure 11**).

## 4 Discussion

On July 26, 2023, doctors and scientists from Stanford University published a groundbreaking study in Nature. This study unveiled a new compound capable of modulating the function of the BCL6 protein, switching its role from blocking gene expression to activating gene expression (16). This discovery opens up new possibilities for developing anticancer drugs targeting BCL6-associated cancers, such as diffuse large B-cell lymphoma. BCL6 plays a crucial role in the differentiation of follicular helper T cells (TFH) and B cells. It is also essential for various processes, such as the formation of BCL6 in the germinal center, maturation of B cell affinity, production of high affinity antibodies, differentiation of plasma cells, and generation of memory B cells. The intricate relationship between follicular helper T cells and tumors warrants further investigation. The number of publications in this field had been steadily increasing since 2012, with a significant surge in recent years. In the past five years, the number of publications had more than doubled compared to previous years, indicating a growing interest among researchers. China led in the number of publications, accounting for nearly half of the global publication volume. The year with the highest number of publications was 2022, with 217 articles (18.37%). Notable authors in this field were Gaulard, Philippe (France), de Léval, Laurence (Switzerland), and Tarte, Karin (France). The most cited authors were Crotty, S (USA), Newman, Am (USA), and Gu Trantien, C (Belgium). Although China had the highest number of publications in the field of TFH cells and tumors, the authors with the most publications and citations were mainly from the United States and France, indicating their high-quality research and leading position in the field. Surprisingly, The top 10 institutions with the highest number of published papers are all located in China. The journal with the most publications was FRONTIERS IN IMMUNOLOGY, covering basic, translational, and clinical immunology. The most cited journal was IMMUNITY. Research on TFH cells and tumor immunity was a current hot topic, reflected in the top 10 cited references. Analysis of keywords revealed ongoing hotspots and research trends, shifting towards “tumor microenvironment”, “tumor infiltrating immune cells”, and “immune infiltration”.

### 4.1 Research focus

TFH cells are crucial mediators in tumor progression and anti-tumor immunotherapy. Previous studies have linked the presence of TFH cells to prognosis in specific cancer types. High levels of TFH cell infiltration have been shown to correlate with enhanced survival rates. Additionally, the efficacy of immune checkpoint inhibitors is influenced by TFH cell activity. Understanding the role and regulation of TFH cells in tumors could pave the way for more effective immunotherapies, ultimately improving survival rates and treatment outcomes for cancer patients. TFH cells express high levels of PD-1 and other co stimulatory and inhibitory receptors. Therefore, treatment targeting CTLA-4 or PD-1 and their ligand PD-L1 may significantly affect TFH cell function in patients receiving immune checkpoint inhibitors(ICIs) therapy, providing a link between ICI therapy and the development of secondary autoimmunity (17). Fudan University’s team led by Xiao Fei has revealed that MCRS1 can enhance the sensitivity of tumor cells to T-cell killing by upregulating MHC-I molecule expression in solid tumors, while improving the therapeutic effect of PD-1 blockade therapy (18). TFH cells have been known to play a crucial role in defending against infectious diseases. For instance, the IgG response to cowpox virus infection is significantly diminished by 98% in the absence of TFH cells (19). Additionally, TFH cells can be targeted and infected by HIV (20). TFH cells are also implicated in autoimmune diseases, such as systemic lupus erythematosus (SLE) and rheumatoid arthritis (RA). The role of TFH cells in human vaccines for annual influenza immunization has been extensively investigated. The role of TFH cells has extended to antibody-mediated allergies. A study in 2012 showed that TFH cell-derived IL-4 is a key factor for inducing IgE production in a mouse model of worm infection (21). Additionally, their role in promoting atherosclerosis has been noted (22). In the context of organ transplantation, TFH cells and GCs present are associated with both acute and chronic rejection reactions in human kidney transplants (23, 24). The last ten years have seen remarkable discoveries linking TFH cells to a broad spectrum of human diseases, particularly cancer. In malignant tumors, the presence of infiltrating immune cells could impact tumor growth, cancer advancement, and patient outcomes (25).

TFH related cells have demonstrated diverse impacts on the long-term survival of patients with different cancer types. For example, in patients with breast cancer or colorectal cancer, TFH cells were positively correlated with survival (26, 27). However, in a mouse model of hepatocellular carcinoma, an inverse correlation between TFH cells and survival was observed (28). Further analysis has revealed that the immune infiltrating components in tumors changed throughout the tumor’s progression, significantly influencing patient survival. Specifically, the density of T follicular helper cells and intrinsic cells tend to increase as the tumor progress, while the density of most T cells decreased. Overall, TFH cells are increasingly recognized for their critical roles across various fields, especially in oncology. TFR (T follicular regulatory cells) are a relatively less mentioned population that may correspond to TFH cells and participate in regulating immune responses, particularly in autoimmune diseases and immune tolerance. The ratio of TFR cells to TFH cells may change in certain disease states, such as in systemic lupus erythematosus (SLE), where low-dose IL-2 therapy can inhibit TFH cells and expand TFR cells, thereby regulating pathogenic humoral immunity (29).

### 4.2 Hotspots and Frontiers

In recent years, research on TFH cells and tumors has shifted towards a deeper understanding of the “tumor microenvironment”, “tumor infiltrating immune cells”, and “immune infiltration”. The field of cancer treatment has significantly evolved over the past decade, moving away from traditional chemotherapies that target a broad spectrum of tumors to new treatment strategies that target cells within the tumor microenvironment. Immune checkpoint blockade therapies, which target immune cells expressing CTLA4,CD28,ICOS and PD1 in the tumor microenvironment, represents a first-generation antibody-based therapy for cancer (30). These inhibitors maintain T cell attack on tumors by inhibiting PD-1 function. We are familiar with PD-1 as an inhibitory receptor expressed on the surface of T cells, which interacts with PD-L1 and PD-L2 (B7-H1 and B7-DC) of the B7 family. PD-1 recruits phosphatases SHP1 and SHP2 through its intracellular tyrosine motif, thereby inhibiting T cell activation. In the tumor immune system, upregulation of PD-1 leads to T cell exhaustion, which is a mechanism of tumor immune escape (31). In addition, CD28 is a co stimulatory molecule belonging to the immunoglobulin superfamily, mainly expressed on all mouse T cells and most human CD4+T cells. CD28 provides the second signal required for T cell activation by binding to CD80 and CD86 (B7 family molecules) on antigen-presenting cells (APCs). The activation of CD28 leads to T cell proliferation and differentiation into various effector cell types. Without co stimulatory signals, T cells may become unresponsive to further stimuli (known as incompetence) or even undergo apoptosis (32). CTLA-4 is a homologous molecule of CD28, primarily expressed on regulatory T cells (Tregs) and upregulated upon activation of conventional T cells. CTLA-4 has a strong inhibitory effect on T cell function, and mice lacking CTLA-4 exhibit lymphoproliferative disorders. CTLA-4 competes with CD28 for CD80/86 on the surface of APCs, thereby controlling T cell activation (32). ICOS is another co stimulatory molecule of the CD28 family, and its ligand is ICOS-L (B7-H2), belonging to the B7 family. The expression of ICOS on the surface of T cells is rapidly upregulated after TCR cross-linking and/or CD28 co stimulation. ICOS-L is expressed on APCs and binding to ICOS triggers intracellular signaling, promoting T cell activation and differentiation (33).

Many researchers now recognize the tumor microenvironment as an active promoter in cancer progression, rather than merely a passive observer. From 2021 to 2022, studies have increasingly focused on how TFH cells interact with the tumor microenvironment and tumor infiltrating immune cells. TFH cells, also known as follicular helper T cells, play a crucial role in the tumor microenvironment and can impact the response to immune therapy and tumor prognosis. Different subpopulations of B cells identified in the tumor microenvironment may exhibit diverse roles, either promoting tumor growth or combating it (34–36). Studies have shown that tumor neoantigens can regulate their fate by promoting the interaction between tumor specific CD4 T cells and tumor specific B cells, thereby enhancing the effector function of CD8 T cells to promote anti-tumor immunity (37). In human colorectal tumors, Overacre Delgoffe et al. investigated the effect of immunogenic bacterium Helicobacter pylori (Hhep) on the immune response to colorectal cancer. Introducing Hhep into a mouse model of colorectal cancer (CRC) increased tumor infiltration of cytotoxic lymphocytes and inhibited tumor growth. Therefore, the introduction of immunogenic intestinal bacteria can promote TFH related anti-tumor immunity in the colon (38). suggesting that TFH cells might enhance the anti-tumor immune response. However, certain tumors may impair TFH cells functions by modifying the tumor microenvironment to evade immune defenses. Laurence Zitvogel et al. proposed a novel coordinated participant that provides humoral and cellular immune responses, operable to restore sensitivity to immune checkpoint inhibition. This operation leads to effective TFH and B cell dialogue in the mesenteric lymph nodes, ultimately resulting in tumor specific memory CD8 T cell responses and preservation of normal epithelium (39). Thus, modulating the function of TFH cells may boost the efficacy of immunotherapies against tumors.

Malignancies can be influenced by the presence of TFH cells, which can either support malignant B cells or provide assistance in combating solid tumors. In tumors with TLS, increasing the number or function of TFH cells may help boost anti-tumor immune responses. Tumors are often infiltrated by various immune cells, such as lymphocytes, macrophages, and mast cells. While lymphocytes can influence cancer outcomes, factors produced by mast cells can promote tumor growth through chronic inflammation (40). Recent research from 2023 to 2024 has focused on the concept of “immune infiltration” involving TFH cells and tumors. TFH cells, a subset of T lymphocytes, play a crucial role in lymphoid tissue and invasive diseases. Their presence in tumor immune infiltration is essential for regulating the immune response of T and B lymphocytes, which ultimately impacts the efficacy of immunotherapies and the prognosis of tumors. Some early studies have mentioned the presence of lymphoid structures in tumors, including breast cancer (41). However, the prognostic value of these structures was demonstrated in non-small cell lung cancer, where the number of mature dendritic cells served as an indicator of tumor lymphoid structures (42). However, the prognostic value of these structures was demonstrated in a study on non-small cell lung cancer, where the number of mature dendritic cells was used as an indicator of tumor lymphoid structures (41, 43). Despite this, their immunological significance in these patients remains unclear. The immune system may struggle to inhibit tumor growth, yet the presence of T follicular helper cells that produce CXCL13 is linked to organized immune structures near the tumor site (44). This association is thought to contribute to sustained and effective long-term anti-tumor immunity. The research group led by Liu Guangwei from the School of Life Sciences at Beijing Normal University has found that the NAD+- dependent deacetylase SIRT3 in mitochondria can regulate the differentiation and function of TFH cells, and play a key regulatory role in anti-tumor immunity. This study provides new experimental evidence for the research of targeted T-cell subpopulation tumor immunotherapy strategies (45). Nowadays, more and more new therapies are being discovered, including dietary fiber promoting cancer immunotherapy by maintaining the microbiota (46);Immune checkpoint inhibitors (ICI) that activate T cells may also lead to AID called rheumatoid immune related adverse events (Rh irAEs) (47); Treat responsive melanoma with ICI (immune checkpoint inhibitors) or MAPK pathway inhibitors (MAPKi) (48); Combination therapy between IL-21 and TFH cells and immune checkpoint in immunotherapy for non-small cell lung cancer (NSCLC) (49); And chimeric antigen receptor (CAR) T-cell therapy for rare cancer such as angioimmunoblastic T-cell lymphoma (AITL) (50). The presence of T follicular helper cells is associated with favorable immune infiltration and tumor prognosis, suggesting a key role in regulating tumor immune response. Enhancing the function of T follicular helper cells could potentially promote immune cell infiltration and elimination of tumors, thereby enhancing tumor immune response.

### 4.3 Advantages and shortcomings

This study conducted a bibliometric analysis of TFH cells and tumors using the Web of Scientific Core Collection (WoSCC). The study identified current research hotspots and trends from various perspectives. However, the study has limitations. Firstly, it included only English-language publications, which may introduce selection bias by excluding literature published in other languages or non-English journals. Secondly, the focus was restricted to articles and review articles, which may lead to incomplete data. In addition, the analysis relies solely on the Web of Science, potentially omitting relevant studies from other databases.

### 4.4 Conclusion

Overall, our bibliometric analysis reveals a rapid growth in research on TFH cells and tumors over the past decade. Since 2012, there has been a surge in interest, with China, the United States, and France emerging as the main contributing countries. Leading the pack in contributions are Shanghai Jiao Tong University, Sun Yat Sen University, and Fudan University. In recent years, “tumor microenvironment”, “tumor infiltrating immune cells”, and “immune infiltration” have emerged as popular research topics that warrant further exploration and attention.

## Data Availability

The raw data supporting the conclusions of this article will be made available by the authors, without undue reservation.
